# Simultaneous Floating-Base Estimation of Human Kinematics and Joint Torques

**DOI:** 10.3390/s19122794

**Published:** 2019-06-21

**Authors:** Claudia Latella, Silvio Traversaro, Diego Ferigo, Yeshasvi Tirupachuri, Lorenzo Rapetti, Francisco Javier Andrade Chavez, Francesco Nori, Daniele Pucci

**Affiliations:** 1 Dynamic Interaction Control at Istituto Italiano di Tecnologia, Center for Robotics and Intelligent Systems, Via San Quirico 19D, 16163 Genoa, Italy; silvio.traversaro@iit.it (S.T.); diego.ferigo@iit.it (D.F.); yeshasvi.tirupachuri@iit.it (Y.T.); lorenzo.rapetti@iit.it (L.R.); franciscoJavier.AndradeChavez@iit.it (F.J.A.C.); francesco.nori@iit.it (F.N.); daniele.pucci@iit.it (D.P.); 2 Machine Learning and Optimisation, The University of Manchester, Manchester M13 9PL, UK; 3 DIBRIS, University of Genova, 16145 Genova, Italy

**Keywords:** floating-base dynamics estimation, human joint torque analysis, human wearable dynamics

## Abstract

The paper presents a stochastic methodology for the simultaneous floating-base estimation of the human whole-body kinematics and dynamics (i.e., joint torques, internal and external forces). The paper builds upon our former work where a fixed-base formulation had been developed for the human estimation problem. The presented approach is validated by presenting experimental results of a health subject equipped with a wearable motion tracking system and a pair of shoes sensorized with force/torque sensors while performing different motion tasks, e.g., walking on a treadmill. The results show that joint torque estimates obtained by using floating-base and fixed-base approaches match satisfactorily, thus validating the present approach.

## 1. Introduction

In physical human–robot interaction (pHRI) domains, a huge variety of applications requires robots to actively collaborate with humans. More and more frequently, robots are required to be endowed with the capability to control physical collaboration through intentional interaction with humans. The simultaneous whole-body estimation of the human kinematics (i.e., motion) and dynamics (i.e., joint torques and internal forces) is a crucial component for modeling, estimating and controlling the interaction. In assistive and rehabilitation scenarios, for instance, the demand for physical robotic assistance to humans is an ever-growing practice and the estimation is a pivotal component for creating technologies capable to help and assist humans. The importance of controlling the pHRI calls for the design of a framework in which the concurrent estimation of the human kinematics and dynamics could be exploited by the robots [1].

In general, several routes for pHRI have been explored over the years. Minimum-jerk-based methods [2,3], imitation learning [4] and retargeting techniques [5,6] are only some of the relevant examples. None of them, however, deal with the simultaneous estimation of the human kinematics and dynamics. Although real-time solutions for whole-body motion tracking are widely marketed (e.g., marker-based motion capture such as Vicon or marker-less systems such as Microsoft Kinect and Xsens wearable suit system), the dynamics estimation is still a challenging problem, especially in those scenarios for which an online estimation is a crucial requirement (e.g., health monitoring or manufacturing ergonomy).

This paper builds upon our former work [7], where a probabilistic algorithm to estimate the human whole-body fixed-base dynamics is described. We propose here a stochastic methodology for the simultaneous *floating-base* estimation of the human kinematics and dynamics. The estimation is computed by means of a sensor-fusion-based tool able to provide an estimation of the whole-body kinematics and dynamics of the human (torques, internal forces exchanged across joints, and external forces acting on links) by leveraging the reliability of the available measurements. The core of the algorithm has been here adapted to a new version to address the need of the floating-base estimation, in which the position and the velocity of the human base are not assumed to be known a priori. The floating-base formalism is not a novelty in the robotics research and it has been used for multiple topics for years. Inverse dynamics control for humanoids and legged robots [8], modeling and control of humanoids in dynamic environments [9], identification of humanoids inertial parameters [10,11], are only a few examples of applications. In [12,13], the formalism has been even used in the context of human–robot experiments. The human dynamics, however, has been not computed under the floating-base formalism but human motion capture data have been used to generate human-like motions to be retargeted onto floating-base humanoids.

Our experimental validation setup considered a sensorized human subject walking on a treadmill. In general, the gait analysis requires: (*i*) classifying the human walking state, i.e., the recursive switching pattern from *right leg single support → double support → left leg single support*; and (*ii*) defining an algorithm able to detect the pattern classification. In a recent study on the human joint muscular torques estimation during gait [14], two dynamical models have been considered separately for the legs to overcome the problem of the switching contact detection and to avoid the increasing complexity of the control algorithm for pattern classification. In this work, we decided to perform a different choice: we considered the dynamics of the human as a whole (with the pelvis as a floating base) and we developed an algorithm to detect the feet contact via additional sensors readings (force/torque sensors). In general, the algorithm constitutes a powerful and versatile tool to arbitrarily analyze all those tasks for which a switching contact condition is required without changing the base inside the algorithm.

The paper is structured as follows. Section 2 defines the mathematical notation and describes the human kinematics and dynamics modeling. Section 3 describes the steps useful to compute the kinematics and joint torques whole-body estimation for the floating-base formalism. In Section 4, the experimental setup and data analysis are described. Conclusions, limitations and several considerations on future developments are discussed in Section 5.

## 2. Background

### 2.1. Notation

- Let R and N be the set of real and natural numbers, respectively.
- Let $x\in R^{n}$ denote a *n*-dimensional column vector, while *x* denotes a scalar quantity. We advise the reader to pay attention to the notation style: we define vectors, matrices with bold small and capital letters, respectively, and scalars with non-bold style.
- Let |x| be the norm of the vector x.
- Let $0_{m}$ and $1_{m}$ be the zero and identity matrices $\in R^{m\times m}$, respectively. The notation $0_{m\times n}$ represents the zero matrix $\in R^{m\times n}$.
- Let I be an inertial frame with *z*-axis pointing against the gravity (*g* denotes the norm of the gravitational acceleration). Let B denote the base frame, i.e., a frame attached to the base link. Let L be the generic frame attached to a link, and J the frame of a joint.
- Let each frame be identified by an origin and an orientation, e.g., $I=(O_{I},\left[I\right])$ or $L[I]=(O_{L},\left[I\right])\;$.
- Let ${}^{I}o_{B}\in R^{3}$ be the coordinate vector connecting $O_{I}$ with $O_{B}$, pointing towards $O_{B}$, expressed with respect to (w.r.t.) frame I.
- Let ${}^{I}R_{B}\in SO\left(3\right)$ be a rotation matrix such that ${}^{I}o_{L}={}^{I}o_{B}+{}^{I}R_{B}\;{}^{B}o_{L}\;$.
- Let S(x)∈SO(3) denote the skew-symmetric matrix such that S(x)y=x×y, being × the cross product operator in $R^{3}$.
- Let ${}^{I}{\dot{o}}_{B}$ and ${}^{I}{\ddot{o}}_{B}$ denote the first-order and second-order time derivatives of ${}^{I}o_{B}\;$, respectively.
- Given a stochastic variable x, let p(x) denote its probability density and p(x|y) the conditional probability of x given the assumption that another stochastic variable y has occurred.
- If E[x] is the expected value of a stochastic variable x, let $\mu_{x}=E\left[x\right]$ and $\Sigma_{x}=E\left[xx^{⊤}\right]$ be the mean and covariance of x, respectively. Let $x∼N(\mu_{x},\Sigma_{x})$ be the expression for the normal distribution of x.

### 2.2. Human Kinematics and Dynamics Modeling

**Figure 1:**
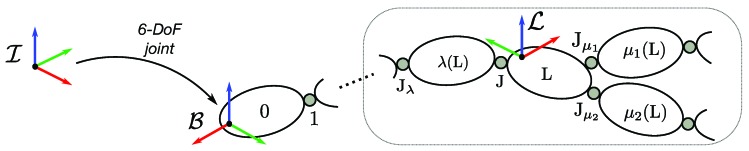
Graphical representation of the system topological order for links and joints.

The human is modeled as a rigid multi-body system with n∈N internal Degrees of Freedom (DoFs). The system is composed of $N_{B}$ rigid bodies, called *links* (denoted with L) connected by *joints* (denoted with J). Links are numbered from 0 to $N_{B}-1$, being 0 the floating base. Furthermore, λ(L) and μ(L) represent the parent and child links of L, respectively. The topological order is such that links L and λ(L) are coupled by joint J. The joint motion constraint is modeled with the motion freedom subspace $S\in R^{6}$. We assume that all the joints have one DoF each, which implies that the joint numbering is from 1 to $n=N_{B}-1$. No parent joint is assumed for the floating base. See the overall representation in Figure 1.

We also assume that none of the links have a known a priori constant pose w.r.t. I. Thus, we say that the system is floating base. More precisely, the system configuration space is a Lie group $Q=R^{3}\times SO\left(3\right)\times R^{n}$ such that $q=(q_{b},s)\in Q$, being $q_{b}=\left({}^{I}o_{B},{}^{I}R_{B}\right)\in R^{3}\times SO\left(3\right)$ the pose of the base frame B w.r.t. I and $s\in R^{n}$ the joint positions vector capturing the topology of the system. The velocity of the system is represented by $\nu =\left({}^{I}v_{B},\dot{s}\right)\in R^{6+n}$ being ${}^{I}v_{B}=\left({}^{I}{\dot{o}}_{B},{}^{I}\omega_{B}\right)\in R^{6}$ the velocity of B w.r.t. I (the angular velocity of the base ${}^{I}\omega_{B}$ is such that ${}^{I}{\dot{R}}_{B}=S\left({}^{I}\omega_{B}\right){}^{I}R_{B}\;$). $\dot{s}\in R^{n}$ is the joint velocities vector. If the system is interacting with the external environment by exchanging $n_{c}$ wrenches, the dynamics of the floating-base system can be described by adopting the Euler–Poincaré formalism ([15], Ch. 13.5):

(1) $$\begin{array}{c} M\left(q\right)\dot{\nu }+h\left(q,\nu \right)=B\tau +\sum_{k=1}^{n_{c}}J_{C_{k}}^{⊤}\left(q\right){𝕗}_{k}^{x}, \end{array}$$

where $M\in R^{\left(n+6)\times (n+6\right)}$ is the mass matrix, $h\in R^{n+6}$ is a vector accounting for the Coriolis effects and gravity terms, $B:={\left(0_{n\times 6},1_{n}\right)}^{⊤}$ is a selector matrix, $\tau \in R^{n}$ represents the joint torques, and ${𝕗}_{k}^{x}=\left(f_{k}^{x},m_{k}^{x}\right)\in R^{6}$ is a vector representing the external wrench acting on the system on the link that has the *k*th contact point, being $f_{k}^{x}$ and $m_{k}^{x}$ the external force and moment, respectively. The Jacobian $J_{C_{k}}\left(q\right)\in R^{6\times (6+n)}$ is an operator that maps the system velocity ν with the velocity v at the *k*th contact frame, such that

(2) $$\begin{array}{c} {{}^{I}v}_{C_{k}}=J_{C_{k}}\left(q\right)\nu =\left[\begin{array}{cc} J_{b}\left(q\right) & J_{s}\left(q\right) \end{array}\right]\left[\begin{array}{c} {{}^{I}v}_{B}\\ \dot{s} \end{array}\right]\;, \end{array}$$

with $J_{b}\left(q\right)\in R^{6\times 6}$ and $J_{s}\left(q\right)\in R^{6\times n}$ Jacobians being related to the base and joint configuration, respectively.

### 2.3. Case-Study Human Model

Our case-study human body owns $N_{B}=67$ links and n=66 internal DoFs. The links were modeled with simple geometric shapes (parallelepiped, cylinder, and sphere) whose dimensions were estimated via inertial measurement units (IMUs) readings (i.e, Xsens motion capture system provides the position of several anatomical bony landmarks w.r.t. the origin of each link). The dynamic properties of each link (i.e., inertias and center of mass) were computed via the anthropometric data available in the literature by: (*i*) exploiting the relation between the total body mass and the mass of each link [16,17]; and (*ii*) assuming geometric approximations and homogeneous density for the links [18,19].

## 3. Simultaneous Floating-Base Estimation of Human Whole-Body Kinematics and Dynamics

In this section, we describe step-by-step the simultaneous floating-base estimation algorithm for the human whole-body kinematics and dynamics.

### 3.1. Offline Estimation of Sensor Position

**Figure 2:**
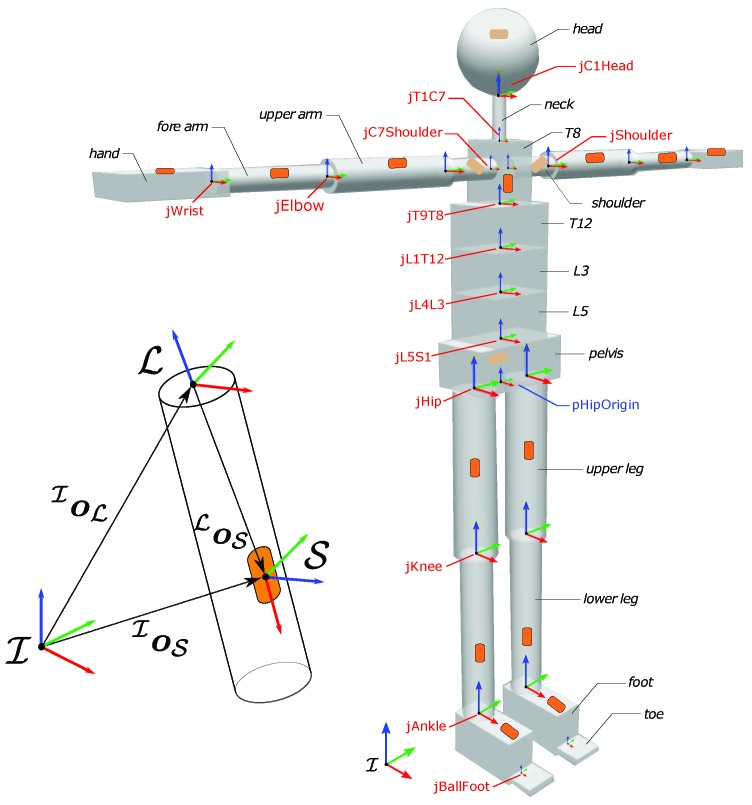
Human model with distributed inertial measurement units. Joint reference frames are shown by using RGB (Red–Green–Blue) convention for *x*–*y*–*z*-axes. (left) Detail of the sensor position on the link.

The first objective was to develop a Universal Robot Description Format (URDF) model for the human with properties listed in Section 2.3 (see Figure 2, right). The URDF is a XML-based file format for representing the kinematics and dynamics of multi-body systems. A crucial step for the URDF generation is to identify the position of each sensor w.r.t. the attached link frame (Figure 2, left). Xsens exposes the sensor linear acceleration, the link angular velocity and acceleration, the sensor and link orientation w.r.t. the inertial frame. However, the sensor position is not provided by its framework. A procedure to estimate the sensor position by processing IMUs data was therefore adopted. The procedure is very similar to the one in [20], where it is used for humanoid robots.

More precisely, if S is the frame associated to the sensor and L is the frame of the link where the sensor is rigidly attached, then the measurement equation is such that

(3) $$\begin{array}{cc} a_{S} & ={}^{S}R_{I}\left({}^{I}{\ddot{o}}_{S}-{}^{I}g\right)\\ & ={}^{S}R_{I}\left[{}^{I}{\ddot{o}}_{L}+{}^{I}{\dot{\omega }}_{L}\times {}^{I}R_{L}\;{}^{L}o_{S}+{}^{I}\omega_{L}\times \left({}^{I}\omega_{L}\times {}^{I}R_{L}\;{}^{L}o_{S}\right)-{}^{I}g\right]\\ & ={}^{S}R_{I}\left[{}^{I}{\ddot{o}}_{L}+\left(S\left({}^{I}{\dot{\omega }}_{L}\right)+S{\left({}^{I}\omega_{L}\right)}^{2}\right){}^{I}R_{L}\;{}^{L}o_{S}-{}^{I}g\right]\;, \end{array}$$

being ${}^{I}g={\left[0\;0-9.81\right]}^{⊤}$.

Equation (3) can be rearranged in the following form:

(4) $$\begin{array}{c} \underset{A}{\underset{︸}{\left(S\left({}^{I}{\dot{\omega }}_{L}\right)+S{\left({}^{I}\omega_{L}\right)}^{2}\right){}^{I}R_{L}}}{}^{L}o_{S}=\underset{b}{\underset{︸}{{}^{I}R_{S}a_{S}-\left({}^{I}{\ddot{o}}_{L}-{}^{I}g\right)}}. \end{array}$$

Given $N_{m}$ measurements, the position of the sensor w.r.t. the link, i.e., ${}^{L}o_{S}$, is the solution of the following optimization problem:

(5) $$Lo_{S}^{*}=\arg\min_{Lo_{S}}\;{\left|\bar{A}Lo_{S}-\bar{b}\right|}^{2}$$

being $\bar{A}={\left[A_{1}\;A_{2}\ldots A_{N_{m}}\right]}^{⊤}$, $\bar{b}={\left[b_{1}\;b_{2}\ldots b_{N_{m}}\right]}^{⊤}$.

### 3.2. Estimation of Human Kinematics

The objective of this section is to derive algorithms for estimating the human kinematic configuration $q=(q_{b},s)$ and its derivatives.

Per each link pair [λ(L),L] coupled by the joint J, $s\in R^{n}$ was computed by solving an optimization problem using Ipopt [21]. The problem is formulated to minimize the distance between the measured, i.e., ${}^{\lambda (L)}R_{L}^{meas}$, and the computed, i.e., ${}^{\lambda (L)}R_{L}$, relative rotations between the frames attached to the link pair, such that

(6) $$s_{J}=\arg\min_{s}\;distance\left({}^{\lambda (L)}R_{L}^{meas}\;,{}^{\lambda (L)}R_{L}\right)\;,\;\;s.t.\;s_{J,min}<s_{J}<s_{J,max}$$

being the distance defined in terms of the rotation error parameterized in Euler angles, and $\left(s_{J,min},s_{J,max}\right)$ joint limits. We refer to Equation (6) as a *link-pairwise* inverse kinematics (IK) problem. Joint velocities and accelerations $\dot{s},\ddot{s}\in R^{n}$ were computed by using a weighted sum of moving windows of elements with a third-order polynomial Savitzky–Golay filtering [22].

The base pose $q_{b}$ was obtained via IMUs readings. The pivotal modification for the floating-base formalism deals with the computation of the velocity ${}^{I}v_{B}$ of the floating base. It is assumed that holonomic constraints in the form of c(q)=0 act on the system in Equation (1). In the human experimental framework, constraints occur when the system is in contact with the ground such that the feet can be considered as end-effectors with zero velocity (i.e., ${{}^{I}v}_{C_{k}}=0$). This yields to

(7) $$\begin{array}{c} 0=J_{C_{k}}\left(q\right)\nu . \end{array}$$

If RF and LF are the contact frames associated to the right and left foot, respectively, for Equation (2), we can write Equation (7) such that

(8a) $$\begin{array}{ccc} 0 & = & J_{b,\mathrm{RF}}\left(q\right)\;{{}^{I}v}_{B}+J_{s,\mathrm{RF}}\left(q\right)\dot{s}\;, \end{array}$$

(8b) $$\begin{array}{ccc} \Leftrightarrow \;{}^{I}v_{B}^{*} & = & \arg\min_{{}^{I}v_{B}}|\;J_{b,\mathrm{RF}}\left(q\right)\;{}^{I}v_{B}+J_{s,\mathrm{RF}}\left(q\right)\dot{s}{|}^{2}\;, \end{array}$$

if only the contact in RF occurs, and

(9a) $$\begin{array}{ccc} 0 & = & J_{b,\mathrm{LF}}\left(q\right)\;{{}^{I}v}_{B}+J_{s,\mathrm{LF}}\left(q\right)\dot{s}\;, \end{array}$$

(9b) $$\begin{array}{ccc} \;\Leftrightarrow \;{}^{I}v_{B}^{*} & = & \arg\min_{{}^{I}v_{B}}|\;J_{b,\mathrm{LF}}\left(q\right)\;{}^{I}v_{B}+J_{s,\mathrm{LF}}\left(q\right)\dot{s}{|}^{2}\;, \end{array}$$

if the contact occurs in LF.

Nevertheless, if the system is simultaneously constrained by both feet, we need to consider the overall effect on the system,

(10a) $$\begin{array}{ccc} 0 & = & \underset{{\bar{J}}_{b(q)}}{\underset{︸}{\left[\begin{array}{c} J_{b,\mathrm{RF}}\left(q\right)\\ J_{b,\mathrm{LF}}\left(q\right) \end{array}\right]}}{}^{I}v_{B}\underset{{\bar{J}}_{b(q)}}{\underset{︸}{\left[\begin{array}{c} J_{s,\mathrm{RF}}\left(q\right)\\ J_{s,\mathrm{LF}}\left(q\right) \end{array}\right]}}\dot{s} \end{array}$$

(10b) $$\;\Leftrightarrow \;{}^{I}v_{B}^{*}=\arg\min_{{}^{I}v_{B}}{\left|{\bar{J}}_{b}\left(q\right){}^{I}v_{B}+{\bar{J}}_{s}\left(q\right)\overset{\cdot }{s}\right|}^{2}$$

### 3.3. Offline Contact Classification

We implemented an offline algorithm to detect which foot is in contact with the ground, i.e., double support state, left single support state, or right single support state (see Algorithm 1). The contact classification is determined via force/torque (FT) sensors readings and depends on a self-tuned threshold force value $T_{f_{z}}$. The threshold defines how big the area of the double support has to be considered. When a single support occurs, the algorithm is able to classify which foot is in contact with the ground by reading and comparing the FT sensors values.

**Algorithm 1** Offline Feet Contact Cassification.**Require:** FT sensor forces (*z* component) for right foot ${}^{RF}f_{z}$ and left foot ${}^{LF}f_{z}$1:
**procedure**
2:    *N* ← number of samples3:    $T_{f_{z}}$ ← threshold on *f_z_* = *mean*(${}^{RF}f_{z}+{}^{LF}f_{z}$)4:    *main loop*:5:    **for**
j=1→N
**do**6:        **if**
$abs\left({}^{RF}f_{z}-{}^{LF}f_{z}\right)\leq T_{f_{z}}$
**then**7:           Classify *j* as double support sample8:        **else**9:           **if**
${}^{RF}f_{z}\left(j\right)>{}^{LF}f_{z}\left(j\right)$  **then**10:               Classify *j* as right single support sample11:           **else**12:               Classify *j* as left single support sample13:           **end if**14:        **end if**15:    **end for**16:  **end procedure**

### 3.4. Maximum-A-Posteriori Algorithm for Floating-Base Dynamics Estimation

The simultaneous estimation of the human kinematics and dynamics is performed by means of a Maximum-A-Posteriori (MAP) algorithm. The advantages of this algorithm are discussed in [7]. Here, the objective is to describe how the core of the algorithm was modified to fit the floating-base formalism. The main difference lies in a new representation for the acceleration. Instead of using the *proper body acceleration* $\in R^{6}$ (e.g., as in [23]), i.e.,

(11) $$\begin{array}{c} a_{L}^{g}={\dot{v}}_{L}-\left[\begin{array}{c} {}^{L}R_{I}{}^{I}g\\ 0_{3\times 1} \end{array}\right]=\left[\begin{array}{c} {}^{I}{\ddot{o}}_{L}\\ {}^{I}{\dot{\omega }}_{L} \end{array}\right]-\left[\begin{array}{c} {}^{L}R_{I}{}^{I}g\\ 0_{3\times 1} \end{array}\right]\;, \end{array}$$

we decided to adopt the *proper sensor acceleration* $\in R^{6}$, i.e.,

(12) $$\begin{array}{ccc} \alpha_{L}^{g} & = & \alpha_{L}-\left[\begin{array}{c} {}^{L}R_{I}{}^{I}g\\ 0_{3\times 1} \end{array}\right]={}^{L}X_{L[I]}{}^{L[I]}{\dot{v}}_{L}-\left[\begin{array}{c} {}^{L}R_{I}{}^{I}g\\ 0_{3\times 1} \end{array}\right]\\ & = & \left[\begin{array}{cc} {}^{L}R_{I} & 0_{3}\\ 0_{3} & {}^{L}R_{I} \end{array}\right]\left[\begin{array}{c} {}^{I}{\ddot{o}}_{L}\\ {}^{I}{\dot{\omega }}_{L} \end{array}\right]-\left[\begin{array}{c} {}^{L}R_{I}{}^{I}g\\ 0_{3\times 1} \end{array}\right]=\left[\begin{array}{c} {}^{L}R_{I}{}^{I}{\ddot{o}}_{L}\\ {\dot{\omega }}_{L} \end{array}\right]-\left[\begin{array}{c} {}^{L}R_{I}{}^{I}g\\ 0_{3\times 1} \end{array}\right]. \end{array}$$

being $X\in R^{6\times 6}$ the adjoint transformation matrix for motion vectors. The main advantage is that the linear part of Equation (12) corresponds to Equation (3) where the frame of the accelerometer coincides with the frame L of the link (same origin and orientation). In general, several modifications were required for the floating-base formalism as follows.

- We used the new acceleration representation by exploiting the relation between Equation (11) and Equation (12), i.e.,
  (13) $$a_{L}^{g}=\alpha_{L}^{g}-{\bar{\alpha }}_{L}^{g}\;\mathrm{being}\;{\bar{\alpha }}_{L}^{g}=\left[\begin{array}{c} \left({}^{L}R_{I}{}^{I}{\dot{o}}_{L}\right)\times \omega_{L}\\ 0_{3\times 1} \end{array}\right]$$
- Since we broke the univocal relation between each link and its parent joint, we redefine the serialization of all the kinematics and dynamics quantities in the vector d w.r.t. the fixed-base serialization of the same vector in Section 4 of [7], thus
  (14) $$\begin{array}{ccc} d & = & {\left[\begin{array}{cc} d_{link}^{⊤} & d_{joint}^{⊤} \end{array}\right]}^{⊤}\in R^{12N_{B}+7n}\;, \end{array}$$
  being
  (15a) $$\begin{array}{ccc} d_{link} & = & \left[\begin{array}{ccccccc} \alpha_{0}^{g} & {𝕗}_{0}^{x} & \alpha_{1}^{g} & {𝕗}_{1}^{x} & \cdots & \alpha_{N_{B}-1}^{g} & {𝕗}_{N_{B}-1}^{x} \end{array}\right]\in R^{12N_{B}}\;, \end{array}$$
  (15b) $$\begin{array}{ccc} d_{joint} & = & \left[\begin{array}{cccccccc} {𝕗}_{1} & {𝕗}_{2} & \cdots & {𝕗}_{n} & {\ddot{s}}_{1} & {\ddot{s}}_{2} & \cdots & {\ddot{s}}_{n} \end{array}\right]\in R^{7n}\;. \end{array}$$
  In the new serialization, $\alpha^{g}\in R^{6}$ is the proper sensor acceleration of Equation (12) and ${𝕗}^{x}\in R^{6}$ is the external wrench acting on each link. Similarly for the joint quantities, $𝕗\in R^{6}$ is the internal wrench (or joint wrench) exchanged from λ(L) to L through the joint J, while $\ddot{s}$ is the joint acceleration.
- The variable τ was removed from d. The joint torque can be obtained as a projection of the joint wrench on the motion freedom subspace, such that $\tau_{J}=S_{J}^{⊤}{𝕗}_{J}$, for each joint J of the model.

Within this new formalism, we rewrite the equation of the acceleration propagation and the recursive Newton–Euler equations. The Newton–Euler formalism is an equivalent representation of Equation (1) (more details about this choice can be found in Section 3.3 of [24]). For the sake of simplicity, here following, we refer to L and J as compact forms for $L_{L}$ and $J_{J}$, respectively, thus:

(16) $$\begin{array}{c} \alpha_{L}^{g}={}^{L}X_{\lambda (L)}{}^{\lambda (L)}\alpha_{\lambda (L)}^{g}+S_{J}{\ddot{s}}_{J}+\left[\begin{array}{c} 0_{3\times 1}\\ \omega_{L} \end{array}\right]\times S_{J}{\dot{s}}_{J}+({\bar{\alpha }}_{L}^{g}-{}^{L}X_{\lambda (L)}{}^{\lambda (L)}{\bar{\alpha }}_{\lambda (L)}^{g}) \end{array}$$

(17) $$M_{L}\alpha_{L}^{g}+\left[\begin{array}{c} 0_{3\times 1}\\ \omega_{L} \end{array}\right]\times {}^{*}M_{L}\left[\begin{array}{c} 0_{3\times 1}\\ \omega_{L} \end{array}\right]={𝕗}_{L}^{x}+{𝕗}_{J}-\sum^{J}X_{L\mu }^{*}{𝕗}_{L\mu },$$

where $X^{*}\in R^{6\times 6}$ and $\times^{*}$ are the adjoint transformation matrix and the dual cross product operator $\in R^{3}$ for force vectors, respectively. In general, these equations seem to be much more complex than the ones obtained by using the proper body acceleration in [7]. They have, however, the convenient property to be agnostic to the linear velocity of each link. This property drastically simplifies the generalization to the floating-base case, in which the linear velocity of the floating base is, in general, not available.

As already described in [24], the estimation problem can be compactly arranged in a matrix form, as follows:

(18) $$\begin{array}{c} \left[\begin{array}{c} Y(s)\\ D(s) \end{array}\right]d+\left[\begin{array}{c} b_{Y}\left(s,\dot{s}\right)\\ b_{D}\left(s,\dot{s}\right) \end{array}\right]=\left[\begin{array}{c} y\\ 0_{12N_{B}\times 1} \end{array}\right]\;. \end{array}$$

More in detail:

- The first set of equations $Y\left(s\right)d+b_{Y}\left(s,\dot{s}\right)=y\;$ accounts for the sensor measurements. The number of equations depends on how many sensors are conveyed into the vector $y\in R^{y}$ and it does not depend on the number of links in the model (more than one sensor could be associated to the same link, e.g., the combination of an IMU + a FT sensor). In general, the sensor matrices are not changed within the new floating-base formalism. The only difference is that the accelerometer has a different relation with the acceleration of the body. In particular, if the frame L of a link and the frame associated to the IMU located on the same link are rigidly connected, then
  (19) $$\begin{array}{c} y_{L,IMU}=\alpha_{IMU}^{g}={}^{L}X_{B}\alpha_{B}^{g}+\left[\begin{array}{c} {}^{L}R_{B}\left[\omega_{B}\times \left(\omega_{B}\times {}^{B}o_{IMU}\right)\right]\\ 0_{3\times 1} \end{array}\right]. \end{array}$$
  Similarly, for the FT sensor frames rigidly connected to the feet frames, the measurement equation is
  (20) $$\begin{array}{c} y_{L,FT}={}^{L}X_{FT}^{*}{𝕗}_{FT}\;. \end{array}$$
- The second set of equations $D\left(s\right)d+b_{D}\left(s,\dot{s}\right)=0\;$ represents the compact matrix form for Equations (16) and (17) given the new serialization of d in Equation (14). The matrix $D\in R^{12N_{B}\times d}$ is a matrix with $12N_{B}$ rows and *d* columns, i.e., the number of rows of d in Equation (14). The matrix blocks in D for the acceleration of Equation (16) are recursively the following:
  (21a) $$\begin{array}{ccc} D_{\alpha_{L}} & = & -1_{6}\;, \end{array}$$
  (21b) $$\begin{array}{ccc} D_{\alpha_{\lambda (L)}} & = & \left\{\begin{array}{cc} {}^{L}X_{\lambda (L)}\in R^{6\times 6} & \forall L\neq B\\ 0_{6} & otherwise \end{array}\right. \end{array}$$
  (21c) $$\begin{array}{ccc} D_{{\ddot{s}}_{J}} & = & \left\{\begin{array}{cc} S_{J}\in R^{6} & \forall L\neq B\\ 0_{6\times 1} & otherwise \end{array}\right. \end{array}$$
  The blocks in D for Newton–Euler equations related to Equation (17) are instead:
  (22a) $$\begin{array}{ccc} D_{\alpha_{L}} & = & M_{L}\in R^{6\times 6}\;, \end{array}$$
  (22b) $$\begin{array}{ccc} D_{f_{L}^{x}} & = & -1_{6}\;, \end{array}$$
  (22c) $$\begin{array}{ccc} D_{f_{J}} & = & \left\{\begin{array}{cc} -1_{6} & \forall L\neq B\\ 0_{6} & otherwise \end{array}\right. \end{array}$$
  (22d) $$\begin{array}{ccc} D_{f_{J_{\mu }}} & = & \left\{\begin{array}{cc} {}^{J}X_{J_{\mu }}^{*}\in R^{6\times 6} & if\;\exists \;\mu (L)\\ 0_{6} & otherwise \end{array}\right. \end{array}$$
  All the other blocks in D are equal to **0**. Unlike D, the term $b_{D}\in R^{12N_{B}}$ is affected by the new representation of the acceleration w.r.t. the one in [7]. Each subterm $b_{D_{L}}\in R^{12}$ is such that
  (23) $$b_{D_{L}}=\{\begin{array}{cc} \left[\begin{array}{c} \;{\bar{\alpha }}_{B}^{g}\\ \left[\begin{array}{c} 0_{3\times 1}\\ \omega_{B} \end{array}\right]\times^{*}M_{B}\left[\begin{array}{c} 0_{3\times 1}\\ \omega_{B} \end{array}\right] \end{array}\right] & if\;L=B\\ \left[\begin{array}{c} \left[\begin{array}{c} \left[\begin{array}{c} 0_{3\times 1}\\ \omega_{L} \end{array}\right]\times S_{J}{\dot{s}}_{J}+\left(\right.{\bar{\alpha }}_{L}^{g}-{}^{L}X_{\lambda (L)}{}^{\lambda (L)}\alpha \end{array}\right]\;{\bar{\lambda }}_{\left(L\right)}^{g}\\ \left[\begin{array}{c} 0_{3\times 1}\\ \omega_{L} \end{array}\right]\times^{*}M_{L}\left[\begin{array}{c} 0_{3\times 1}\\ \omega_{L} \end{array}\right] \end{array}\right] & otherwise \end{array}$$

The solution of the system in Equation (18) is computed in a Gaussian domain via MAP estimator. Within this framework, ***d*** and ***y*** are stochastic variables with Gaussian distributions and the problem is solved by maximizing the conditional probability of d given the measurements y, i.e.,

(24) $$d_{MAP}=\arg\max_{d}p\left(d|y\right).$$

Equation (24) corresponds to the mean of the conditional probability $\mu_{d|y}$, such that

(25a) $$\begin{array}{ccc} \Sigma_{d|y} & = & {\bar{\Sigma }}_{D}^{-1}+Y^{⊤}\Sigma_{y}^{-1}Y^{-1}, \end{array}$$

(25b) $$\mu_{d|y}=\Sigma_{d|y}[Y^{⊤}\Sigma_{y}^{-1}(y-b_{Y})+{\bar{\Sigma }}_{D}^{-1}{\bar{\mu }}_{D}].$$

More details on the MAP solution are provided in Appendix A.

## 4. Experiments and Analysis

### 4.1. Experimental Setup

**Figure 3:**
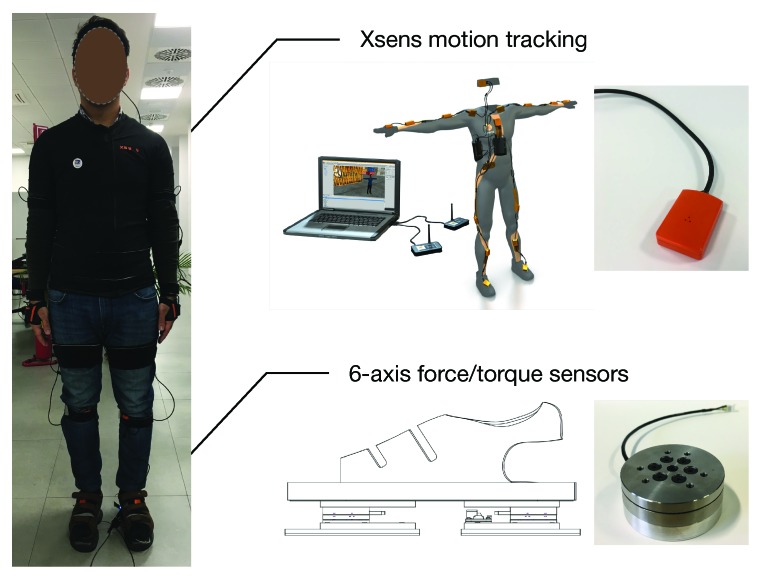
Subject equipped with the Xsens wearable motion tracking system and six-axis force/torque shoes.

The objective of the experiment was to test the goodness of the estimation algorithm. An experimental session was carried out at Istituto Italiano di Tecnologia (IIT), Genoa, Italy, with a healthy male subject. The participant was equipped with an Xsens wearable motion tracking system with 17 IMUs to capture the whole-body kinematics. A pair of sensorized shoes developed at IIT was used to detect the ground reaction forces. Each shoe was equipped with two six-axis FT sensors able to measure 6D wrenches (3 forces and 3 moments), as shown in Figure 3. The subject was asked to perform a set of different tasks, as listed in Table 1.

Data were recorded at 50 Hz via a YARP-based [25] framework for wearable sensors that allows synchronously collecting data coming from multiple sources (see open-source code available on Github repository https://github.com/robotology-playground/wearables). Data processing was analyzed on MathWorks MATLAB^®^. The MAP computation code (open-source at https://github.com/claudia-lat/MAPest) relies on the C++ based iDynTree multi-body dynamics library designed for free floating robots [26]. iDynTree is released as open source code available on Github: https://github.com/robotology/idyntree. Furthermore, it is worth remarking that an important modification on the IK computation was here introduced w.r.t. the one in [7]. We removed the OpenSim IK toolbox dependency and we computed the whole-body joint angles with an Ipopt-based IK (see Section 3.2).

**Figure 4:**
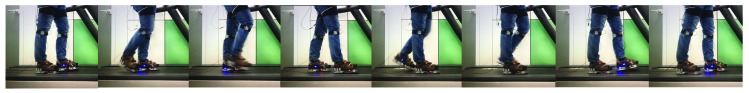
Task T4, Sequence 2: walking on a treadmill.

**Figure 5:**
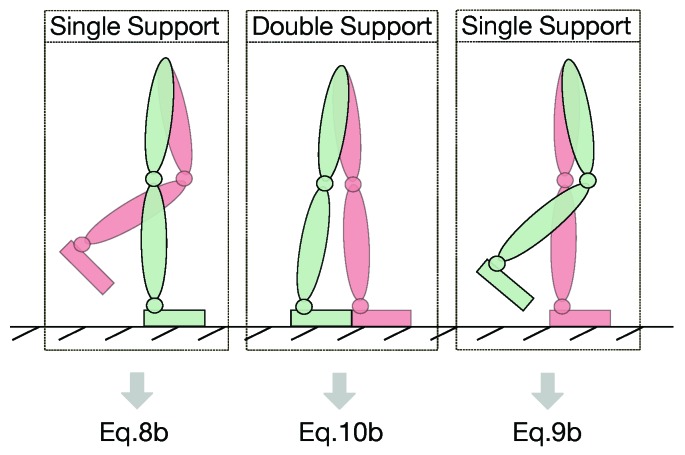
Classification of feet contacts: (**left**) single support on the right foot; (**middle**) double support; and (**right**) single support on the left foot.

**Figure 6:**
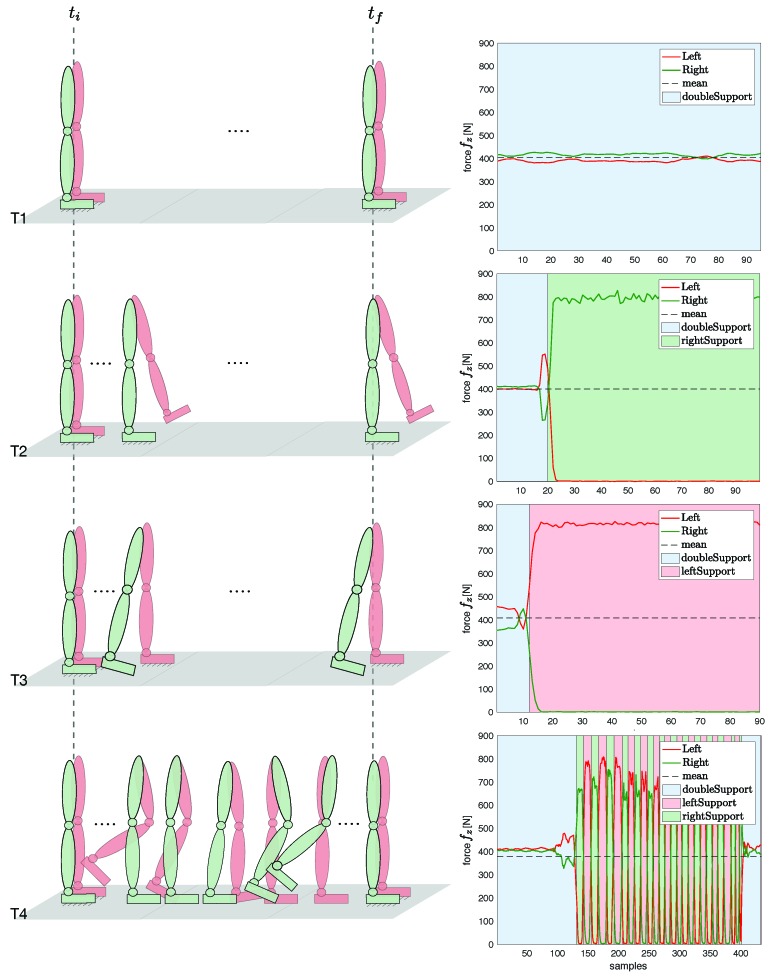
Tasks representation from initial time $t_{i}$ to final time $t_{f}$ (**left**); and feet contact pattern classification obtained via Algorithm 1 (**right**).

Data coming from the shoes FT sensors were analyzed to detect the feet contact, as described in Algorithm 1 in Section 3.3. The overall estimation considered thus Equation (10b) for T1, Equation (8b) for Sequence 2 of T2, and Equation (9b) for Sequence 2 of T3. In Sequence T4 on Figure 4, for instance, the algorithm detected the switching contact condition of the feet in Figure 5 and applied the proper base velocity computation. Figure 6 shows the feet contact detection for the tasks in Table 1.

### 4.2. Comparison between Measurement and Estimation

**Figure 7:**
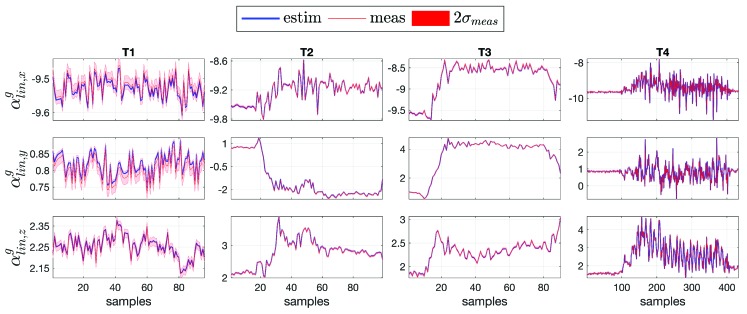
The base linear proper sensor acceleration $\alpha_{lin}^{g}$ [$m/s^{2}$] comparison between measurement (mean and standard deviation, in red) and floating-base MAP estimation (mean, in blue), for tasks T1, T2, T3 and T4.

**Figure 8:**
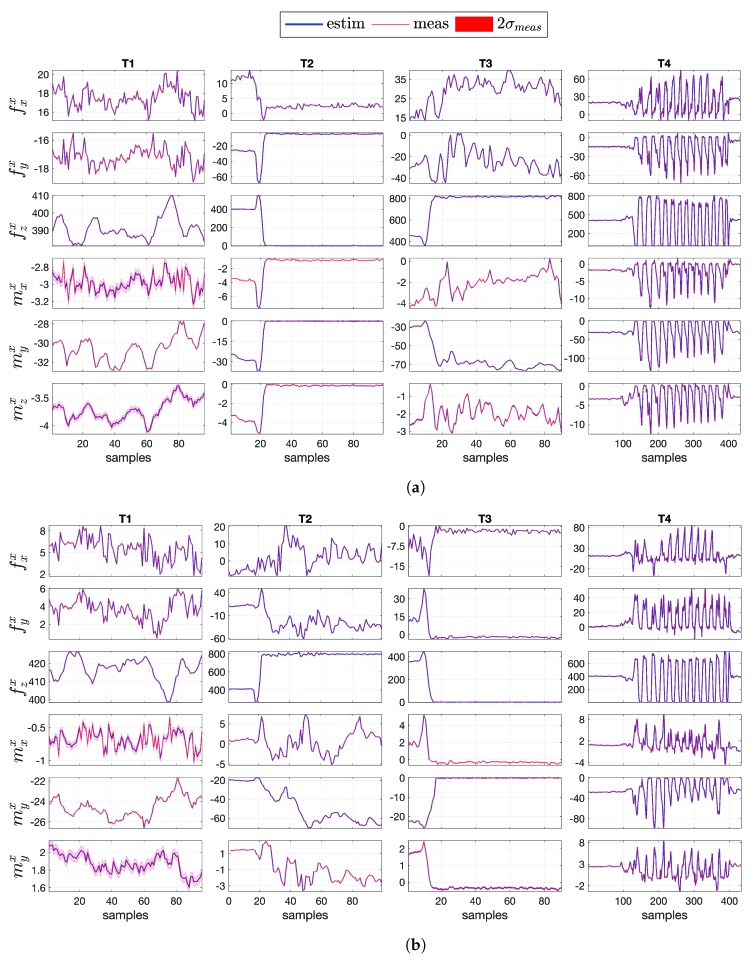
The external force $f^{x}$ [N] and moment $m^{x}$ [Nm] comparison between measurement (mean and standard deviation, in red) and estimation via floating-base MAP algorithm (mean, in blue) for (**a**) the left foot and (**b**) the right foot, respectively, for tasks T1, T2, T3 and T4.

The primary objective of the floating-base MAP algorithm is to estimate simultaneously kinematic and dynamic quantities related to the links and the joints of the human model. The vector ***d*** in Equation (14) contains variables measurable via sensors ($\alpha_{lin}^{g}$, $f^{x}$ and $m^{x}$) and variables that cannot be measured in humans (𝕗 and τ) but only estimated with the algorithm. The MAP algorithm represents the probabilistic way to estimate those quantities for which a quantitative measure does not exist. The objective is to compare the same variables (measured and estimated) to prove the goodness of the proposed algorithm. Figure 7 shows the comparison for the base linear proper sensor acceleration $\alpha_{lin}^{g}$ [$m/s^{2}$] between the measurement (mean and standard deviation, in red) and the estimation via algorithm (mean, in blue), for tasks T1, T2, T3 and T4, respectively. The same comparison for the external force $f^{x}$[N] and external moment $m^{x}$ [Nm] is shown in Figure 8a,b for the left foot and right foot, respectively. The validation was performed along with a Root Mean Square Error (RMSE) investigation for linear accelerations and external wrenches (Table 2). Error range values are shown in Table 3.

It is worth remarking on the importance of the choice of the covariance matrix associated to the sensors. It can be manually tuned as a parameter of the measurement trust. In this experimental analysis, covariances were chosen in a range from $10^{-6}$ to $10^{-4}$: the higher the level of sensor trust (i.e., low covariance), the lower the RMSE associated to the sensor variable.

**Figure 9:**
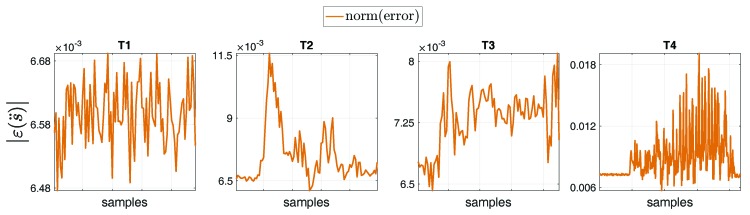
Norm of the overall error of the entire set of joint accelerations $\varepsilon (\ddot{s})$ [$\mathrm{rad}/s^{2}$] between measurement and estimation via floating-base MAP algorithm, for tasks T1, T2, T3 and T4.

Figure 9 represents the norm of the overall error of the joint acceleration $\ddot{s}$ [$\mathrm{rad}/s^{2}$], for tasks T1, T2, T3 and T4. The error norm $\left|\varepsilon \left(\ddot{s}\right)|=|\left({\ddot{s}}_{measured}-{\ddot{s}}_{estimated}\right)\right|$ was computed by considering the entire set of joints of the model, such that $|\varepsilon_{T1}\left(\ddot{s}\right)|=0.007\pm 4.9\times 10^{-5}$ [$\mathrm{rad}/s^{2}$], $|\varepsilon_{T2}\left(\ddot{s}\right)|=0.008\pm 0.001$ [$\mathrm{rad}/s^{2}$], $|\varepsilon_{T3}\left(\ddot{s}\right)|=0.007\pm 3.5\times 10^{-4}$ [$\mathrm{rad}/s^{2}$], $|\varepsilon_{T4}\left(\ddot{s}\right)|=0.009\pm 0.002$ [$\mathrm{rad}/s^{2}$].

### 4.3. Human Joint Torques Estimation during Gait

**Figure 10:**
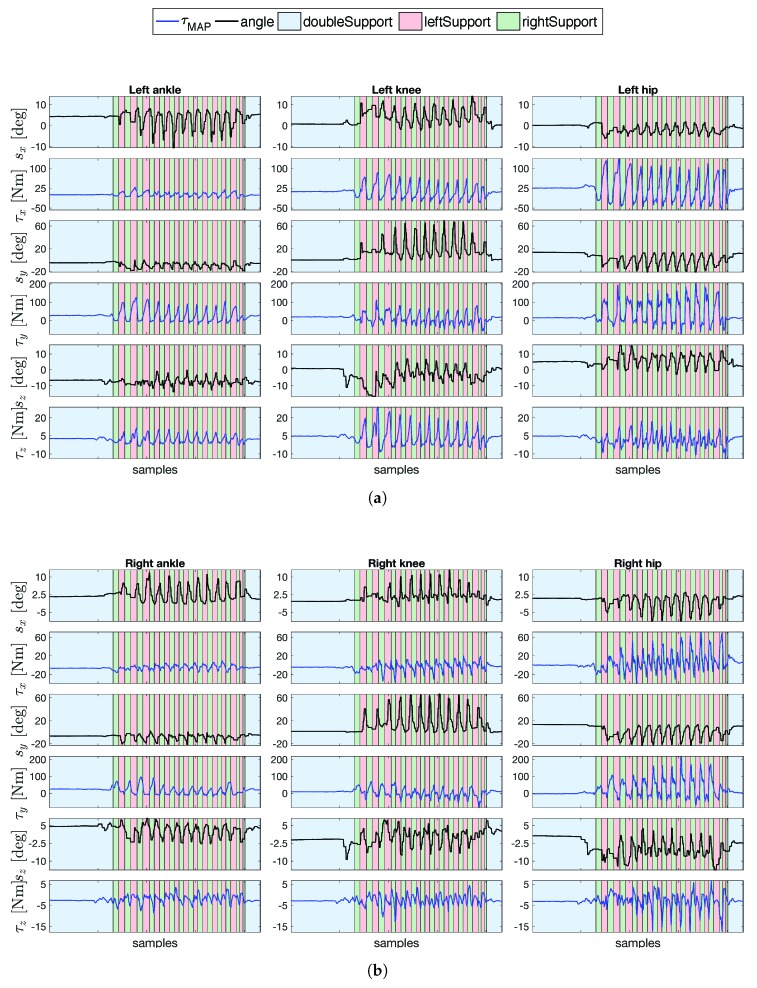
Floating-base MAP estimation in task T4 of the joint torques τ [Nm] (in blue) for the (**b**) left leg and (**a**) right leg, respectively, along with the related joint angles s [deg] (in black). Gait estimations were performed by following the procedure for the feet contact classification in Algorithm 1.

The floating-base MAP algorithm provides the whole-body joint torque estimation. The estimated torque does not have a measured quantity to be compared. We can trust only the estimation as a consequence of the validation analysis in Section 4.2. The algorithm becomes particularly useful when dealing with the human gait analysis. Figure 10 shows the joint torque estimations along with the joint angles for the ( Figure 10a) left leg and ( Figure 10b) right leg, respectively. We decided, here, to show only the most representative results for the walking task.

### 4.4. Comparison between Fixed-Base and Floating-Base Algorithms

**Figure 11:**
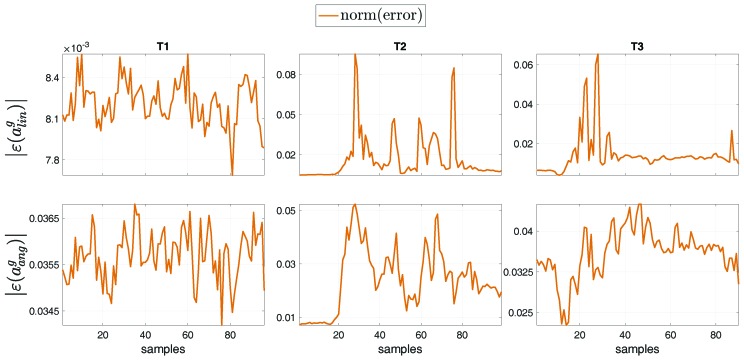
Norm of the error of the base proper body linear acceleration $\varepsilon (a_{lin}^{g})$ [$m/s^{2}$] and angular acceleration $\varepsilon (a_{ang}^{g})$ [$\mathrm{rad}/s^{2}$] between the estimation with the fixed-base and the floating-base MAP, for tasks T1, T2 and T3. The proper body acceleration for the floating-base estimation is obtained via Equation (13).

**Figure 12:**
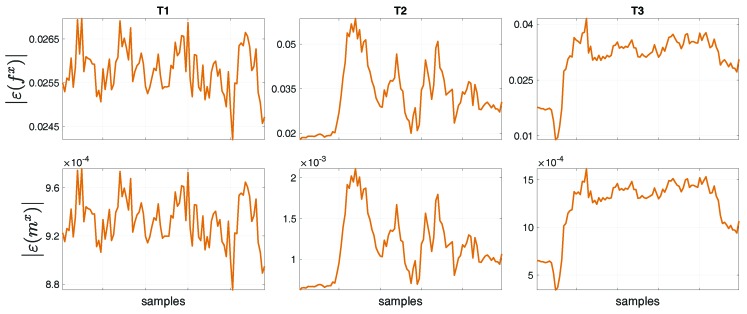
Norm of the error of the overall set of the overall external forces $\varepsilon (f^{x})$ [N] and the moments $\varepsilon (m^{x})$ [Nm] between the estimation with the fixed-base and the floating-base MAP, for tasks T1, T2 and T3.

**Figure 13:**
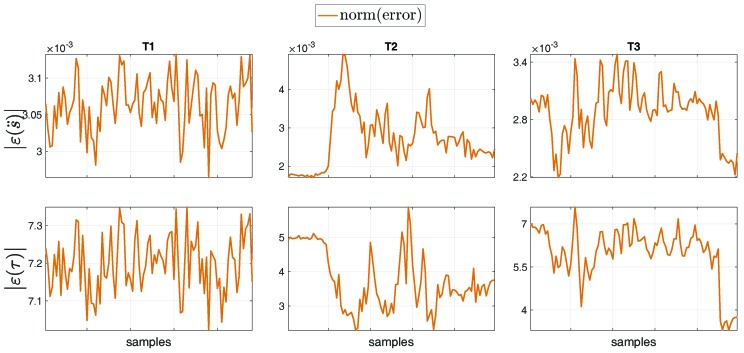
Norm of the error of the overall set of joint accelerations $\varepsilon (\ddot{s})$ [$\mathrm{rad}/s^{2}$] and torques ε(τ) [Nm] between the estimation with the fixed-base and the floating-base MAP, for tasks T1, T2 and T3.

We performed a comparison between the floating-base estimation w.r.t. the fixed-base estimation done in [24] by computing the norm of the error between the two formalisms, for tasks T1, T2 and T3. In general, the error norm was computed as the norm of the difference between each fixed-base and the floating-base estimated variable, i.e, $\left|\varepsilon \left(variable\right)|=|\left(variable_{estimatedFixed}-variable_{estimatedFloating}\right)\right|$. Figure 11 shows the norm of the error for the base proper body linear acceleration $\varepsilon (a_{lin}^{g})$ [$m/s^{2}$] and angular acceleration $\varepsilon (a_{ang}^{g})$ [$\mathrm{rad}/s^{2}$] of the base (i.e, the Pelvis) between the estimations with the formalisms. The proper body acceleration for the floating-base MAP estimation was obtained via Equation (13). The same comparison was performed for the overall set of external force $\varepsilon (f^{x})$ [N] and moment $\varepsilon (m^{x})$ [Nm] errors (Figure 12), and for the entire set of joint acceleration $\varepsilon (\ddot{s})$ [$\mathrm{rad}/s^{2}$] and torque ε(τ) [Nm] (Figure 13) errors. Table 4 shows the mean and standard deviation of the error norms.

In addition to the analytical modifications for the floating-base formalism described in Section 3.4, there is another important advantage in using the floating-base MAP. Unlike the fixed-base estimation where it is fundamental to change the base among the tasks (left foot for tasks T1 and T3, and right foot for T2), this limitation does not exist for the floating-base algorithm, e.g., the pelvis remains the base for all the tasks. Furthermore, the floating-base formalism allows us to make up for the lack of the external force estimation that exists for the fixed-base algorithm on the link appointed as the model base.

### 4.5. A Word of Caution on the Covariances Choice

The MAP algorithm estimation depends on the covariance values chosen by the end-user. In Equations (25a) and (25b), it is visible the role of: (*i*) the measurements covariance $\Sigma_{y}$; and (ii) a covariance ${\bar{\Sigma }}_{D}$ that, in turn, takes into account the model reliability (via covariance $\Sigma_{D}$) and the prior on the estimation (via covariance $\Sigma_{d}$) (see details in Appendix A, Equations (A5a) and (A5b)). In general, the procedural approach consists in assigning

- *low values* for the covariance $\Sigma_{y}$ if trusting in the sensor measurements;
- *low values* for the model covariance $\Sigma_{D}$ for trusting the dynamic model; and
- *high values* for the covariance $\Sigma_{d}$, which means that the end-user does not know any a priori information on the estimation.

The combined contribution of this set of covariances affects the final estimation. The estimation vector d contains variables that are also measured (e.g., linear acceleration, external wrench, and joint acceleration) for which the covariance of the measurement $\Sigma_{y}$ plays a predominant role (a minor role is due to the covariance $\Sigma_{d}$ of the prior). A problem arises, however, when considering the angular acceleration $\alpha_{ang}^{g}$. This variable is part of the d vector as estimation, but it is not measured. At the current stage, a way to play with the angular acceleration trust is, therefore, to tune $\Sigma_{d}$. A forthcoming investigation will deal with the possibility of integrating the angular acceleration as part in the vector y of the measurements.

## 5. Conclusions

In this paper, we present a stochastic methodology for the simultaneous floating-base estimation of the whole-body human kinematics and dynamics (joint torques, internal forces and external forces). The novelty consists in the possibility to perform the estimation in a floating-base framework. The floating base can be arbitrarily chosen among the model links and the algorithm requires to estimate the pose and 6D velocity of the base w.r.t. the inertial frame.

The algorithm was validated by carrying out a four-task experimental session with a healthy subject equipped with a wearable motion tracking system—to capture the whole-body kinematics—and a pair of force/torque sensorized shoes. We performed the tasks in Table 1 by considering the human pelvis as the system (floating) base. This choice came particularly in handy with the walking task on the treadmill. In general, the algorithm allows analyzing all those tasks for which a switching contact condition is implicitly required (e.g., the human gait) without manually tuning the model base into the algorithm.

Current limitations of the methodology concern: (*i*) the human URDF generation and the estimation of sensors position w.r.t. the attached link (see Section 3.1); and (*ii*) the feet contact classification (see Section 3.3), as they are carried out in an offline post-processing step. The impeding objective is to develop a new online procedure to automatize the human model generation from real-time acquisitions together with a real-time algorithm to classify the feet contact. These two features will strongly improve the already existing tool for the online estimation of the human joint torques (open-source code available on Github repository https://github.com/robotology/human-dynamics-estimation).

## Figures and Tables

**Table 1:** Tasks performed for the estimation analysis.

Task	Type	Description
T1	Static double support	Neutral pose, standing still
T2	Static right single support	Sequence 1: static double support
		Sequence 2: weight balancing on the right foot
T3	Static left single support	Sequence 1: static double support
		Sequence 2: weight balancing on the left foot
T4	Static-walking-static	Sequence 1: static double support
		Sequence 2: walking on a treadmill (Figure 4)
		Sequence 3: static double support

**Table 2:** RMSE analysis of the base linear proper sensor acceleration $\alpha_{lin}^{g}$ [$m/s^{2}$], the external force $f^{x}$ [N] and moment $m^{x}$ [Nm] floating-base algorithm estimations w.r.t. the measurements, for tasks T1, T2, T3 and T4.

Task	Link	$\alpha_{\mathrm{lin},x}^{g}$ [m/s^2^]	$\alpha_{\mathrm{lin},y}^{g}$ [m/s^2^]	$\alpha_{\mathrm{lin},z}^{g}$ [m/s^2^]	$f_{x}^{x}$ [N]	$f_{y}^{x}$ [N]	$f_{z}^{x}$ [N]	$m_{x}^{x}$ [Nm]	$m_{y}^{x}$ [Nm]	$m_{z}^{x}$ [Nm]
	Base (Pelvis)	0.008	0.014	0.002	-	-	-	-	-	-
T1	Left foot	-	-	-	0.050	0.030	$2.5\times 10^{-4}$	$8.3\times 10^{-4}$	0.002	$2.1\times 10^{-5}$
	Right foot	-	-	-	0.031	0.048	0.004	0.0015	0.001	$1.0\times 10^{-5}$
	Base (Pelvis)	0.003	0.027	0.018	-	-	-	-	-	-
T2	Left foot	-	-	-	0.153	0.071	0.009	0.002	$4.7\times 10^{-4}$	$1.7\times 10^{-4}$
	Right foot	-	-	-	0.013	0.074	0.005	0.002	$4.2\times 10^{-4}$	$4.3\times 10^{-5}$
	Base (Pelvis)	0.012	0.007	0.007	-	-	-	-	-	-
T3	Left foot	-	-	-	0.075	0.019	0.002	$6.0\times 10^{-4}$	0.002	$5.2\times 10^{-5}$
	Right foot	-	-	-	0.065	0.018	0.003	$6.1\times 10^{-4}$	0.002	$1.2\times 10^{-4}$
	Base (Pelvis)	0.011	0.018	0.033	-	-	-	-	-	-
T4	Left foot	-	-	-	0.089	0.056	0.012	0.002	0.003	$1.3\times 10^{-4}$
	Right foot	-	-	-	0.084	0.056	0.019	0.002	0.003	$9.7\times 10^{-4}$

**Table 3:** Max and min values for RMSE analysis in Table 2, for tasks T1, T2, T3 and T4.

Variables	T1		T2		T3		T4	
	min	max	min	max	min	max	min	max
$\alpha_{lin,x}^{g}$	0.006	0.0086	$2.0\times 10^{-5}$	0.0087	0.0029	0.0187	$1.6\times 10^{-7}$	0.035
$\alpha_{lin,y}^{g}$	0.012	0.015	0.008	0.053	$1.3\times 10^{-5}$	0.0214	$1.3\times 10^{-4}$	0.055
$\alpha_{lin,z}^{g}$	$1.9\times 10^{-4}$	0.003	0.0062	0.026	0.0015	0.0165	$1.3\times 10^{-5}$	0.147
$f_{LF,x}^{x}$	0.045	0.054	0.0016	0.030	0.0071	0.0893	0.0016	0.318
$f_{LF,y}^{y}$	0.026	0.0329	0.0317	0.1258	$6.2\times 10^{-4}$	0.042	0.0012	0.144
$f_{LF,z}^{z}$	$6.7\times 10^{-6}$	$4.8\times 10^{-4}$	$2.1\times 10^{-4}$	0.021	$5.4\times 10^{-5}$	0.0034	$1.0\times 10^{-5}$	0.094
$m_{LF,x}^{x}$	$7.3\times 10^{-4}$	$9.5\times 10^{-4}$	$9.1\times 10^{-4}$	0.004	$2.3\times 10^{-8}$	0.0014	$1.4\times 10^{-5}$	0.0047
$m_{LF,x}^{y}$	0.0015	0.0013	$2.1\times 10^{-5}$	$9.4\times 10^{-4}$	$3.0\times 10^{-4}$	0.0029	$1.6\times 10^{-5}$	0.010
$m_{LF,x}^{z}$	$1.7\times 10^{-5}$	$2.5\times 10^{-5}$	$4.6\times 10^{-5}$	$3.0\times 10^{-4}$	$3.3\times 10^{-5}$	$7.9\times 10^{-5}$	$1.5\times 10^{-7}$	$7.8\times 10^{-4}$
$f_{RF,x}^{x}$	0.026	0.0362	$3.2\times 10^{-4}$	0.038	0.0066	0.077	$1.4\times 10^{-5}$	0.296
$f_{RF,y}^{y}$	0.045	0.051	0.033	0.1261	$1.5\times 10^{-4}$	0.040	$4.3\times 10^{-6}$	0.141
$f_{RF,z}^{z}$	0.0036	0.0045	0.0015	0.014	$7.3\times 10^{-5}$	0.0061	$5.5\times 10^{-5}$	0.113
$m_{RF,x}^{x}$	0.0014	0.0016	$9.8\times 10^{-4}$	0.004	$1.5\times 10^{-5}$	0.0014	$4.6\times 10^{-6}$	0.005
$m_{RF,x}^{y}$	$9.5\times 10^{-4}$	0.0013	$2.6\times 10^{-6}$	0.0012	$3.3\times 10^{-4}$	0.0029	$2.6\times 10^{-5}$	0.0097
$m_{RF,x}^{z}$	$6.2\times 10^{-6}$	$1.4\times 10^{-4}$	$4.0\times 10^{-6}$	$6.7\times 10^{-5}$	$7.0\times 10^{-6}$	$1.7\times 10^{-4}$	$5.9\times 10^{-5}$	$3.0\times 10^{-4}$

**Table 4:** Mean and standard deviation of the error norm for: (i) the base proper body linear acceleration related to Figure 11; (ii) the external wrench related to Figure 12; and (iii) the joint acceleration and torque related to Figure 13, for tasks T1, T2 and T3.

Error Norm		T1	T2	T3
$\left|\varepsilon (a_{lin}^{g})\right|$	[$m/s^{2}$]	$0.008\pm 1.6\times 10^{-4}$	0.0170±0.0176	0.014±0.010
$\left|\varepsilon (a_{ang}^{g})\right|$	[$\mathrm{rad}/s^{2}$]	$0.0357\pm 5.4\times 10^{-4}$	0.024±0.012	0.036±0.005
$\left|\varepsilon (f^{x})\right|$	[N]	$0.026\pm 5.3\times 10^{-4}$	0.033±0.010	0.031±0.006
$\left|\varepsilon (m^{x})\right|$	[Nm]	$9.330e^{-4}\pm 1.9\times 10^{-5}$	$0.001\pm 3.8\times 10^{-4}$	$0.001\pm 3.0\times 10^{-4}$
$\left|\varepsilon (\ddot{s})\right|$	[$\mathrm{rad}/s^{2}$]	$0.003\pm 3.7\times 10^{-5}$	$0.003\pm 7.4\times 10^{-4}$	$0.003\pm 3.0\times 10^{-4}$
|ε(τ)|	[Nm]	7.199±0.076	3.756±0.845	5.988±0.943

## Appendix A

Appendix A provides the reader with a description of the Maximum-A-Posteriori (MAP) estimator as a tool for the whole-body kinematics and dynamics human estimation. More details can be found in [24].

The problem in Equation (18) is here treated in a Gaussian domain. Let ***d*** and ***y*** be stochastic variables with Gaussian distributions. The conditional probability of ***d*** given ***y*** is

(A1) $$p\left(d|y\right)=\frac{p(d,y)}{p(y)}=\frac{p(d)p(y|d)}{p(y)}\;\propto p\left(d\right)p\left(y|d\right)\;,$$

since the term p(y) does not depend on d. The solution provided by the MAP estimator yields to

(A2) $$d_{MAP}=\arg\max_{d}p\left(d|y\right)\;\propto \arg\max_{d}\;p\left(d,y\right)\;.$$

The solution consists in the computation of p(y|d), p(d) and then of p(d|y).

- The conditional probability p(y|d) is:
  (A3) $$\begin{array}{ccc} p(y|d) & \propto & \exp-\frac{1}{2}\left\{{\left(y-\mu_{y|d}\right)}^{⊤}\Sigma_{y|d}^{-1}\left(y-\mu_{y|d}\right)\right\}\\ & = & \exp-\frac{1}{2}\left\{{\left[y-\left(Yd+b_{Y}\right)\right]}^{⊤}\Sigma_{y|d}^{-1}\left[y-\left(Yd+b_{Y}\right)\right]\right\}, \end{array}$$
  which implicitly makes the assumption that the set of measurements equations $Y\left(s\right)d+b_{Y}\left(s,\dot{s}\right)=y$ is affected by a Gaussian noise with zero mean and covariance $\Sigma_{y|d}$.
- Let $d∼N({\bar{\mu }}_{D},{\bar{\Sigma }}_{D})$ the normal distribution of ***d***. The probability density *p*(***d***)
  (A4) $$p\left(d\right)\propto \exp-\frac{1}{2}\left\{{\left(d-{\bar{\mu }}_{D}\right)}^{⊤}{\bar{\Sigma }}_{D}^{-1}\left(d-\mu {\bar{\mu }}_{D}\right)\right\},$$
  where the covariance and the mean are, respectively,
  (A5a) $${\bar{\Sigma }}_{D}={\left(D^{⊤}\Sigma_{D}^{-1}D+\Sigma_{d}^{-1}\right)}^{-1}$$
  (A5b) $${\bar{\mu }}_{D}={\bar{\Sigma }}_{D}(\Sigma_{d}^{-1}\mu_{D}-D^{⊤}\Sigma_{D}^{-1}b^{D})$$
  In particular, covariances $\Sigma_{D}$ and $\Sigma_{d}$ account for the reliability of the model constraints and on the estimation prior, respectively, in the equation $D\left(s\right)d+b_{D}\left(s,\dot{s}\right)=0$.
- To compute p(d|y), it suffices to combine Equations (A3) and (A4),
  (A6) $$\begin{array}{c} p\left(d|y\right)\propto \exp-\frac{1}{2}\left\{{\left(d-{\bar{\mu }}_{D}\right)}^{⊤}{\bar{\Sigma }}_{D}^{-1}\left(d-{\bar{\mu }}_{D}\right)+{\left[y-(Yd+b_{Y})\right]}^{⊤}{\bar{\Sigma }}_{y|d}^{-1}\left[y-(Yd+b_{Y})\right]\right\} \end{array}$$
  with covariance matrix and mean as follows:
  (A7a) $$\begin{array}{ccc} \Sigma_{d|y} & = & {\bar{\Sigma }}_{D}^{-1}+Y^{⊤}\Sigma_{y|d}^{-1}Y^{-1}, \end{array}$$
  (A7b) $$\mu_{d|y}=\Sigma_{d|y}\left[Y^{⊤}\Sigma_{y|d}^{-1}\left(y-b_{Y}\right)+{\bar{\Sigma }}_{D}^{-1}{\bar{\mu }}_{D}\right].$$
  In the Gaussian domain, the MAP solution coincides with the mean in Equation (A7b) yielding to:
  (A8) $$\begin{array}{c} d_{MAP}=\mu_{d|y}. \end{array}$$
